# Opioid-Induced Mitogen-Activated Protein Kinase Signaling in Rat Enteric Neurons following Chronic Morphine Treatment

**DOI:** 10.1371/journal.pone.0110230

**Published:** 2014-10-10

**Authors:** Celine Duraffourd, Erica Kumala, Laura Anselmi, Nicholas C. Brecha, Catia Sternini

**Affiliations:** 1 CURE Digestive Diseases Research Center, Division of Digestive Diseases and Department of Medicine, David Geffen School of Medicine, University of California Los Angeles, Los Angeles, California, United States of America; 2 CURE Digestive Diseases Research Center, Division of Digestive Diseases and Department of Neurobiology, David Geffen School of Medicine, University of California Los Angeles, Los Angeles, California, United States of America; 3 Veteran Administration Greater Los Angeles Healthcare System, Los Angeles, California, United States of America; Temple University School of Medicine, United States of America

## Abstract

Opioids, acting at μ opioid receptors, are commonly used for pain management. Chronic opioid treatment induces cellular adaptations, which trigger long-term side effects, including constipation mediated by enteric neurons. We tested the hypothesis that chronic opioid treatment induces alterations of μ opioid receptor signaling in enteric neurons, which are likely to serve as mechanisms underlying opioid-induced constipation. In cultured rat enteric neurons, either untreated (naïve) or exposed to morphine for 4 days (chronic), we compared the effect of morphine and DAMGO (D-Ala2,MePhe4,Gly-ol5 enkephalin) on μ opioid receptor internalization and downstream signaling by examining the activation of the mitogen-activated protein kinase/extracellular signal-regulated kinases 1 and 2 (MAPK/ERK) pathway, cAMP accumulation and transcription factor cAMP Response Element-Binding protein (CREB) expression. μ opioid receptor internalization and MAPK/ERK phosphorylation were induced by DAMGO, but not morphine in naïve neurons, and by both opioids in chronic neurons. MAPK/ERK activation was prevented by the receptor antagonist naloxone, by blocking receptor trafficking with hypertonic sucrose, dynamin inhibitor, or neuronal transfection with mutated dynamin, and by MAPK inhibitor. Morphine and DAMGO inhibited cAMP in naïve and chronic enteric neurons, and induced desensitization of cAMP signaling. Chronic morphine treatment suppressed desensitization of cAMP and MAPK signaling, increased CREB phosphorylation through a MAPK/ERK pathway and induced delays of gastrointestinal transit, which was prevented by MAPK/ERK blockade. This study showed that opioids induce endocytosis- and dynamin-dependent MAPK/ERK activation in enteric neurons and that chronic morphine treatment triggers changes at the receptor level and downstream signaling resulting in MAPK/ERK-dependent CREB activation. Blockade of this signaling pathway prevents the development of gastrointestinal motility impairment induced by chronic morphine treatment. These findings suggest that alterations in μ opioid receptor downstream signaling including MAPK/ERK pathway in enteric neurons chronically treated with morphine contribute to the development of opioid-induced constipation.

## Introduction

The long-term use of opioids for the treatment of moderate to severe pain is a common clinical practice and has increased exponentially during the past decade in the United States [1]. Opioid drugs exert their pharmacological effects by interacting with the opioid receptors (ORs), mostly targeting the μORs. Binding of opioids with μORs in neurons of the central nervous system (CNS) induces analgesia, whereas activation of μORs in neurons of the enteric nervous system (ENS) inhibits gastrointestinal (GI) motility and secretion [2]–[5]. Unfortunately, the beneficial, analgesic effect of opioids decreases with time due to tolerance [6], [7], thus requiring increasing concentrations of opioids for pain control [1]. This intensifies the inhibitory effects of opioids in the GI tract, resulting in the development of opioid bowel dysfunction, a serious condition characterized by abdominal pain and constipation [1], [8], which significantly hampers opioid treatment. Opioid-induced constipation is the most common adverse GI effect resulting from chronic opioid exposure, with a prevalence of 41% in non-cancer patients up to 95% in cancer-patients [9], [10]. Unlike analgesia, the constipating effect of chronic opioid treatment does not subside with time and it is likely that the different outcome of prolonged stimulation in CNS and ENS neurons is due to different signaling of μORs in distinct neuronal populations.

Activated μORs undergo desensitization due to β-arrestin interaction and G proteins uncoupling, dynamin-dependent internalization and resensitization [7]. μORs signal by coupling to inhibitory Gi/Go proteins and activating several effectors, including inhibition of cAMP, modulation of cation channels and activation of mitogen-activated protein kinase (MAPK) pathway to induce a functional response, which results in inhibition of cellular activity by reducing transmitter release [4], [7]. Opioids can be distinguished by their internalization profiles and downstream effectors, which reflect functional selectivity at μOR or ligand-directed signaling [6], [7]. Indeed, many opioid drugs (e.g. fentanyl, etorphine) and endogenous opioids are efficient in internalizing and desensitizing μORs in multiple cell types, whereas morphine does not [6], [11]–[13]. However, morphine can induce desensitization in the absence of internalization [14] and trigger μOR internalization in certain opioid-responsive CNS neurons [15]. Interestingly, in enteric neurons, chronic *in vivo* treatment with morphine enhances the ability of acute morphine to internalize μORs through a dynamin-mediated pathway [16]. Internalization and desensitization are critical events regulating downstream signaling and receptor function, and alteration of these regulatory processes induced by long-term opioid treatment results in intracellular adaptations underlying the development of opioid-induced side effects [6]. In addition, differences in μOR downstream signaling with chronic opioid treatment have been described. Indeed, chronic opioid stimulation results in up-regulation of the cAMP pathway through activation of Gβγ proteins, whereas acute μOR activation inhibits cAMP through activation of Gα protein. cAMP superactivation together with persistent activation of the MAPK cascade that has also been reported in chronically treated neurons represent intracellular adaptations underlying the development of long-term treatment side effects [7]. Opioid-induced constipation results from disruption of GI motility mediated by μORs in enteric neurons [4], [5], [17], thus an elucidation of μOR signaling in these neurons is essential to understand the mechanisms underlying the development of GI side effects such as motility impairment following chronic opioid treatment. μOR signaling is regulated by multiple factors including the type of opioid agonist, and magnitude and length of stimulation, which induce compensatory changes in signal transduction [6]. However, little is known about μOR signaling in enteric neurons. In this study, we tested the hypothesis that μOR agonists differing in their efficiency to induce receptor internalization in enteric neurons [12], [13], differentially regulate MAPK in enteric neurons untreated (naive) or chronically treated with morphine (chronic), and that chronic morphine modulates downstream events mediating alterations of GI motility. We focused on extracellular signal-regulated kinase 1 and 2 (ERK1/2) members of the MAPK family, which are upregulated in different neuronal populations by chronic morphine and produce long-lasting changes [7], [18], [19]. We found that DAMGO, an internalizing opioid, but not morphine, a poor internalizing agonist, induces MAPK/ERK phosphorylation in naïve enteric neurons, whereas both DAMGO and morphine activate MAPK/ERK pathway in chronic enteric neurons. By contrast, opioids inhibit cAMP signaling in acute and chronic neurons irrespective of their internalization efficiency. Chronic morphine triggers activation of the transcription factor, cAMP Response Element-Binding protein (CREB) via a MAPK-dependent pathway and suppresses opioid-induced μOR desensitization. Finally, blockade of MAPK/ERK pathway reversed the delay of GI transit induced by chronic morphine. These findings support the concept that changes in μOR downstream signaling in enteric neurons induced by chronic morphine treatment are important factors underlying the development of long-term GI opioid effects.

## Materials and Methods

Five to 10 day-old (Harlan Labs, San Diego, CA) and adult (200–225 g from Charles River Laboratory International, Inc., Hollister, CA) Sprague Dawley rats were used for this study. Pups and adult rats were housed in rooms with controlled temperature and light-dark cycles. Adult rats had unrestricted access to standard rat chow and tap water and acclimated for 1 week before experimentation.

### Ethics Statement

Animal care and procedures were in accordance with the National Institutes of Health recommendations for the humane use of animals. Experimental procedures were reviewed and approved by the Animal Research Committee of the University of California, Los Angeles (ARC protocol # 2002-012). All efforts were made to minimize the number of animals used and their suffering.

### Preparation of primary cultures of enteric neurons

Primary cultures of myenteric neurons were prepared as described [20]. Pups were euthanized by isoflurane (5%; Phoenix Pharmaceuticals Inc, Burlingame, CA) followed by decapitation. The small intestine was removed, and the longitudinal muscle with attached the myenteric plexus was separated from the whole thickness of the specimen and processed for enzymatic and mechanical dissociation. Neurons were plated at 2.5×10^5^ cells/mL on cover slips coated with poly-L-Lysine (Sigma-Aldrich, St Louis, MO) in DMEM supplemented with 100 U/mL penicillin, 100 U/mL streptomycin (Invitrogen, Carlsbad, CA) and 10% fetal bovine serum (FBS, Life Technologies, Grand Island, NY) at 37°C for 4 hours. Cover slips were placed cell side facing down, which reduces oxygen tension environment and proliferation of non-neuronal cells, in new wells containing Neurobasal-A medium (Invitrogen) with 1% FBS, penicillin, streptomycin and B27-supplement (Invitrogen).

### Effects of μOR-agonists on ERK pathway and μOR internalization

We used the endogenous opioid analogue, DAMGO (D-Ala2,MePhe4,Gly-ol5 enkephalin; Sigma-Aldrich), which is a potent internalizing agonist, and morphine (Sigma-Aldrich), which is a poor internalizing opioid in enteric neurons [13], [16]. Cultured enteric neurons were exposed to medium (naïve neurons) or medium with 10 µM morphine for 4 days (chronic neurons) following plating. Medium was changed every 2 days. On day 5, cover slips were rotated so that cells were facing up and incubated at 37°C for 1 hour in agonist- and serum-free Neurobasal-A medium, then treated with DAMGO (1 µM), morphine (10 µM) or medium alone for 5, 10, 20, and 30 minutes at 37°C. Initial dose-response study tested DAMGO 10 nM–10 µM and morphine 100 nM–100 µM and showed that 1 and 10 µM, respectively were the lowest doses with the highest effect on ERK1/2, thus were selected for subsequent experiments.

To confirm that ERK1/2 activation is receptor mediated and is not due to endogenous opioid release, cells were incubated with the opioid receptor antagonist, naloxone (Sigma-Aldrich, St Louis, MO; 10 µM) for 1 hour or a blocker of endogenous neurotransmitter release, tetrodotoxin (TTX, Sigma-Aldrich; 10 µM) for 1.5 hours prior to opioid stimulation. Enteric neurons were pre-incubated with MEK inhibitor, U0126 (1,4-diamino-2,3-dicyano-1, 4-bis[2-aminophenylthio]butadiene; Cell Signaling, Danvers, MA; 10 µM), which blocks the MAPK cascade leading to ERK, for 1.5 hours [21]. To establish whether opioids induce desensitization, we treated enteric neurons for 2 hours with 1 µM DAMGO or 10 µM morphine, washed and then exposed them to a second stimulation with the same dose of agonist for 5 minutes.

To determine whether DAMGO and morphine induced μOR internalization in cultured enteric neurons, we exposed either naïve or chronic enteric neurons to DAMGO (1 µM), morphine (10 µM) or medium alone for 1 hour at 4°C to allow ligand-receptor binding, then transferred to ligand-free medium at 37°C for 30 minutes to allow receptor internalization and processed for immunohistochemistry [13], [16]. Briefly, cultured neurons were fixed in 4% paraformaldehyde in 0.1 M phosphate buffer (PB), pH 7.4 for 2 hours, washed and incubated in rabbit polyclonal, affinity-purified μOR antibody (Incstar Science, Technology and Research, Stillwater, MN; 1∶3,000) directed to the C-terminus region of rat μ-OR (384–398) in 0.1 M PB/0.5% Triton X-100 (Sigma-Aldrich), and 10% goat serum (Invitrogen, Carlsbad, CA) (overnight at 4°C) followed by Alexa Fluor 488 affinity-purified donkey anti-rabbit (1∶1000; Invitrogen Molecular Probes, Eugene, OR) (1 hour at room temperature). The μOR antibody has been previously characterized [13], [16], [17]. μOR immunoreactivity was visualized using a Zeiss 510 META laser scanning upright compound confocal microscope (Axioplan 2) using the 488 nm line of the argon laser. All images were acquired with a PlanApo 63×1.4 NA objective. Images were processed and labeled using Adobe Photoshop 7.0 (Adobe Systems, Mountain View, CA).

To determine the involvement of μOR internalization in opioid-induced ERK1/2 phosphorylation, enteric neurons were pre-incubated with an hypertonic sucrose solution (Sigma-Aldrich) for 1 hour to block clathrin-coated pits formation. Since upregulation of dynamin facilitates the endocytosis of morphine-activated μORs in chronic enteric neurons [16], we tested whether dynamin plays a role in opioid-induced ERK1/2 activation. We treated enteric neurons for 15 minutes with dynasore (Calbiochem, San Diego, CA; 10 µM), a cell-permeable inhibitor of dynamin [22], prior to opioid stimulation. We also transfected enteric neurons with a mutated dynamin (K44E-dynamin, mutation leading to lack of GTP activity [23], [24], provided by Dr. Bunnett, Monash University, Australia) or wild type (WT) dynamin using the lentiviral transfection method.

### Lentivirus WT or mutated K44E-dynamin constructs and transfection of enteric neurons

WT and mutated rat dynamin sequences were amplified from rat dynamin-HA WT pIRES2 GFP or rat dynamin-HA K44E pIRES2 GFP plasmids by PCR. PCR products were cloned into plasmid pRRL-sin-cPPT-CMV-MCS-IRES-GFP encoding a HIV-derived lentiviral vector with a cytomegalovirus (CMV) promoter to drive high gene expression. Lentivirus-based vectors encoding different isoforms of dynamin were generated by transient co-transfection of 293T cells with a three-plasmid combination [25]. Briefly, 100 mm dishes of nonconfluent 293T cells were co-transfected with 6.5 µg of packaging construct pMDLg/pRRE, 3.5 µg of pMDG (construct encoding the VSV-G envelope), 2.5 µg of pRSV–REV and 10 µg of pRRL-sin-cPPT-CMV-dynamin-IRES-GFP, by CaPi-DNA co-precipitation method. The following day, cells were incubated for 8 hours in 10 mM sodium butyrate to obtain high-titer virus production [25]. Conditioned medium was harvested 16 hours later and passed through 0.45 µm filters. Viral titer was determined by assessing viral p24 antigen concentration by ELISA (Alliance HIV-I p24 ELISA Kit, Perkin Elmer). Transfections were carried out in 1 mL of DMEM, including serial dilutions of lentiviral vector supernatant and 1 mg/mL of protamine sulfate. One day post-transfection, cells were incubated in Neurobasal-A medium complemented with 1% FBS, 100 U/mL penicillin, 100 U/mL streptomycin and B27-supplement for 3 days, then treated with either 1 µM DAMGO, 10 µM morphine, or medium for 5 minutes. Dynamin expression was measured by Western blot (see below) using anti-dynamin 1 antibody (C-16, sc-6402, Santa Cruz), which recognizes the C-terminal part of the protein. This antibody detects both the WT and mutated form of dynamin, since the mutation appears in the 44th amino acid corresponding at the GTPase activity domain, present in the N-terminal part of the protein [23], [24].

### Dynamin, ERK and CREB Western blot assays

Cells were extracted using Laemmli buffer, separated by SDS-polyacrylamide gel electrophoresis and transferred onto PVDF membranes. Membranes were blocked 1 hour at room temperature in LI-COR Blocking buffer (LI-COR Biosciences, Lincoln, NE) and incubated at 4°C overnight with rabbit anti-dynamin 1 antiserum (1∶200), mouse monoclonal antibody to phospho-p44/42 (Thr202/Tyr204) ERK1/2 (pERK1/2) (1∶1000; Cell Signaling, Danvers, MA) or anti phosphorylated-CREB 1 (pCREB) antibody (Ser 133; 1∶1000; Cell Signaling). Total ERK (tERK) or CREB (tCREB) were determined after stripping and reprobing the membranes previously incubated with pERK1/2 or pCREB with rabbit polyclonal anti tERK1/2 (C14) (1∶500; Santa Cruz Biotechnology, CA) or anti tCREB (1∶1000; Cell Signaling), respectively. Antibodies recognizing total proteins (ERK and CREB) were used to verify that the cell treatments did not affect the total level of these proteins and to confirm equal gel loading [26]. To verify equal loading of the gels for dynamin Western blot, a mouse antibody against the housekeeping protein, glyceraldehyde-3-phosphate dehydrogenase (GAPDH), was used (1∶000; Cell Signaling). Immunoreactive bands were visualized by using infrared fluorescent secondary antibodies IRDye 800 Goat anti Mouse (1∶10000) for pERK and GAPDH, and IRDye 680 Goat anti Rabbit (1∶10000) for tERK quantification; and IRDye 800 Goat anti Rabbit (1∶10000) for tCREB, pCREB and dynamin. Images were collected using the LI-COR Odyssey infrared imaging system and staining intensities were quantified with Odyssey Application Software Version 3.0. Values were expressed as a ratio of control at 0 minute.

### cAMP assay

A cAMP assay was used to determine whether morphine induces desensitization in naïve neurons and whether chronic morphine induces cAMP superactivation, a hallmark of chronic opioids treatment [7]. Briefly, naïve or chronically treated enteric neurons were exposed to forskolin (10 µM) and IBMX 0.5 mM for 15 min at 37°C, before addition of opioids, or control solution for 15 min and intracellular cAMP was measured using the Amersham cAMP Biotrak Enzyme Immunoassay System [27].

### Chronic treatment with morphine and assessment of gastrointestinal transit

To determine whether disruption of μOR signaling affects GI transit delay in animal chronically treated with morphine, we used a MEK1/2 inhibitor *in vivo* and measured total GI transit following chronic morphine treatment with or without MEK1/2 inhibitor. Rats received one of the following treatment: saline, MEK1/2 inhibitor (40 mg/kg) [28], morphine (day 1–2, 10 mg/kg; day 3–4, 20 mg/kg; days 5–6, 40 mg/kg) or morphine and MEK1/2 inhibitor twice a day subcutaneously for 6 days (n = 5 per group). GI transit was measured as previously described by evaluating the distribution of non-absorbable fluorescent 70,000 MW dextran (150 µL, 5 mg/mL) administered intragastrically by gavage [29]. Animals received their last injection of saline or drugs 30 minutes after gavage, then they were euthanized by an overdose of isoflurane (5%) followed by thoracotomy. The entire GI tract was excised and divided into 15 segments: stomach, small intestine (10 segments of equal length), cecum and colon (3 segments of equal length). The luminal content of each segment was collected, suspended in 1 mL of distilled water, clarified by centrifugation (15 min at 14,000 rpm at 4°C). The supernatants of individual samples were collected and the fluorescence signal was determined in triplicates by using a fluorescence plate reader (excitation wavelength 485 nm and emission wavelength 528 nm, FLX800 Microplate Fluorescence Reader, Bio-Tek instruments Inc., Winooski, VT). The averaged fluorescent intensity of each segment in triplicate and the sum of all fluorescent averages were measured. The GI transit was expressed as the geometric center (GC) of the distribution of labeled fluorescent dextran along the entire GI tract using the following equation: GC = sum of the products of the fraction of the marker in each segment times the segment number [30].

### Statistical analysis

Data are expressed as mean ± SEM. Statistical differences were analyzed by a one-way ANOVA with Bonferroni post-tests. A p<0.05 was considered significant. Statistical analyses were performed using Prism 5.0 software (GraphPad Software, San Diego, CA).

## Results

### Ligand-directed μOR internalization in cultured enteric neurons

**Figure 1:**
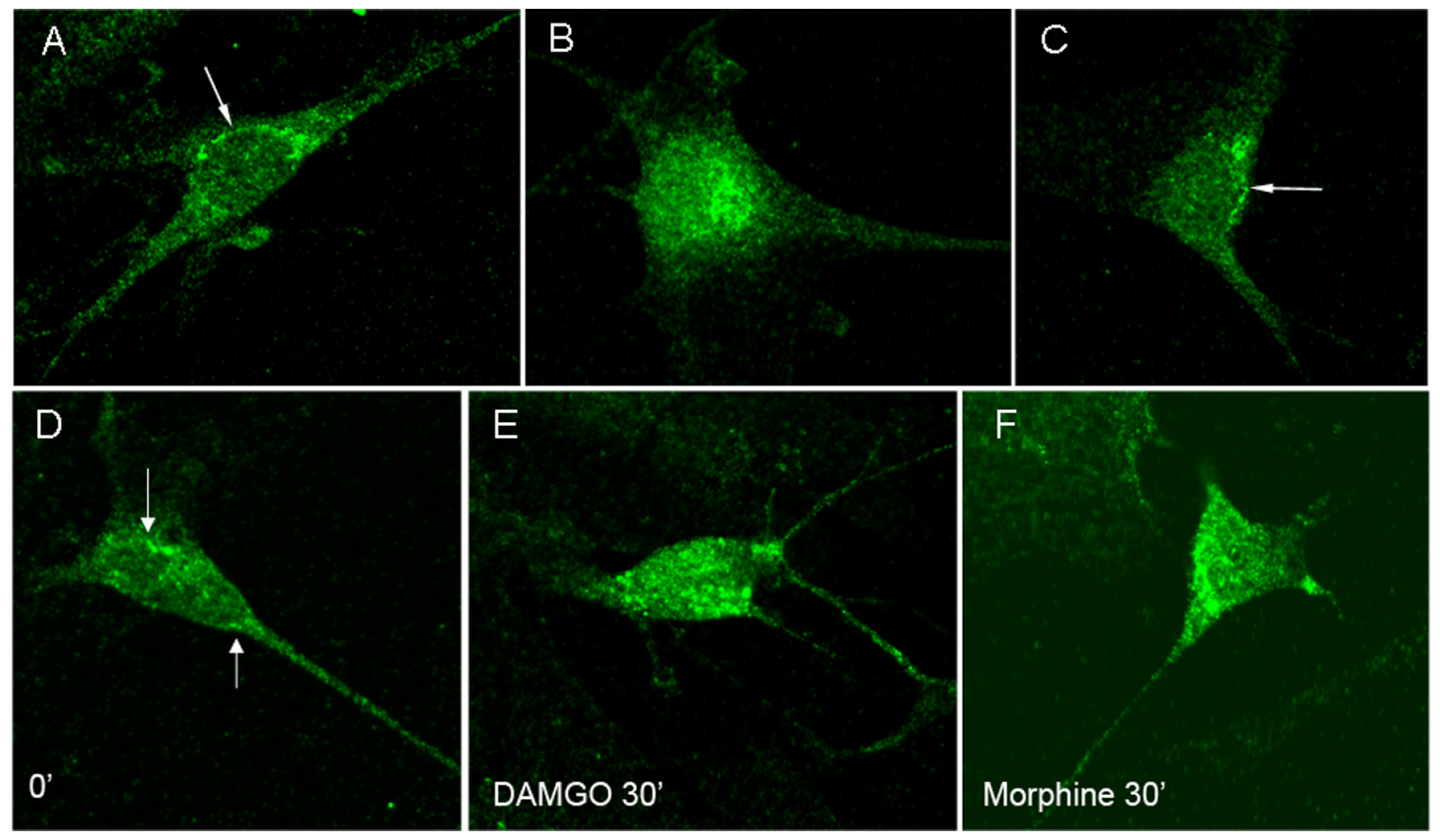
μOR immunoreactivity in naïve enteric neurons (A–C) and in neurons chronically treated with morphine (D–F). μOR immunoreactivity is at the cell surface in unstimulated and morphine-stimulated neurons (A, C arrows), and it is in the cytosol following stimulation with DAMGO (B) in naïve enteric neurons. μOR immunoreactivity is at the cell surface in unstimulated neurons (D, arrows), but in the cytosol following DAMGO or morphine stimulation (E, F) in chronic enteric neurons.

DAMGO, but not morphine induced rapid μOR internalization in cultured naïve enteric neurons, whereas both DAMGO and morphine induced comparable μOR translocation in chronically treated neurons (Figure 1). These results are comparable to published data *in vivo* and *in vitro* [12], [13], [16].

### Effects of DAMGO and morphine on ERK activation in enteric neurons

**Figure 2:**
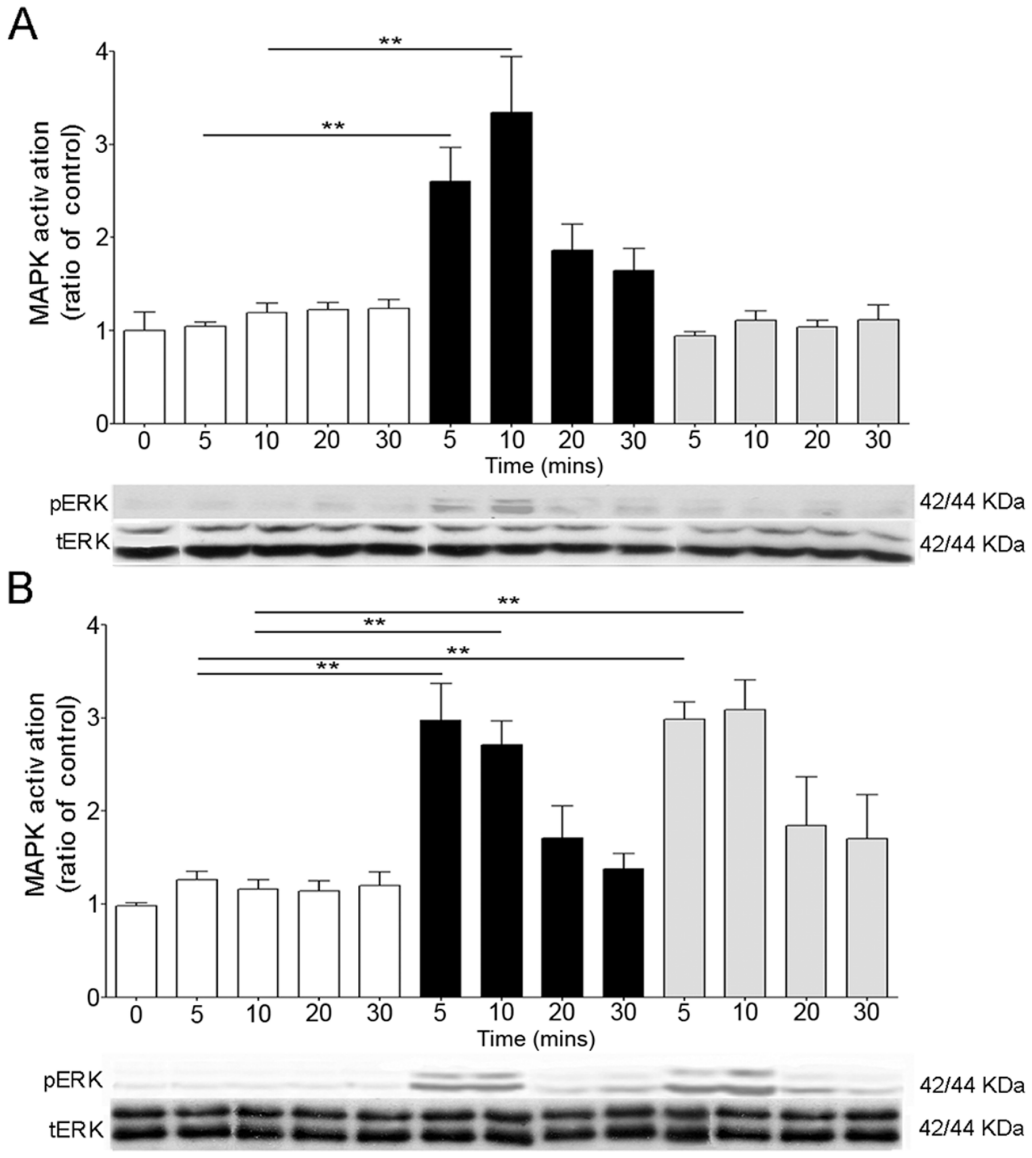
Opioid-induced MAPK activation in naïve (A) and chronically treated (B) enteric neurons. DAMGO (1 µM, black bars) induced a transient MAPK/ERK1/2 activation in naïve (A) and chronic (B) neurons at 5 and 10 minutes, whereas morphine (grey bars) induced MAPK/ERK1/2 activation only in chronic (B) neurons. **p<0.01 compared to controls (white bars). N = 4–7 experiments in triplicate. Representative gels of pERK1/2 and tERK are shown at the bottom of each graph. tERK was used to verify that the treatment did not affect the total level of this protein and to confirm equal gel loading.

In naïve neurons, DAMGO induced a marked and significant increase of ERK1/2 phosphorylation with peaks at 5 and 10 minutes (p<0.01 vs. controls) and returned to baseline at 20 minutes (Figure 2). By contrast, ERK1/2 phosphorylation levels in naïve neurons exposed to morphine were similar to controls (Figure 2), suggesting ligand selectivity of ERK1/2 activation. However, in neurons treated chronically with morphine, both DAMGO and morphine induced significant ERK phosphorylation at 5 and 10 minutes (p<0.01).

**Figure 3:**
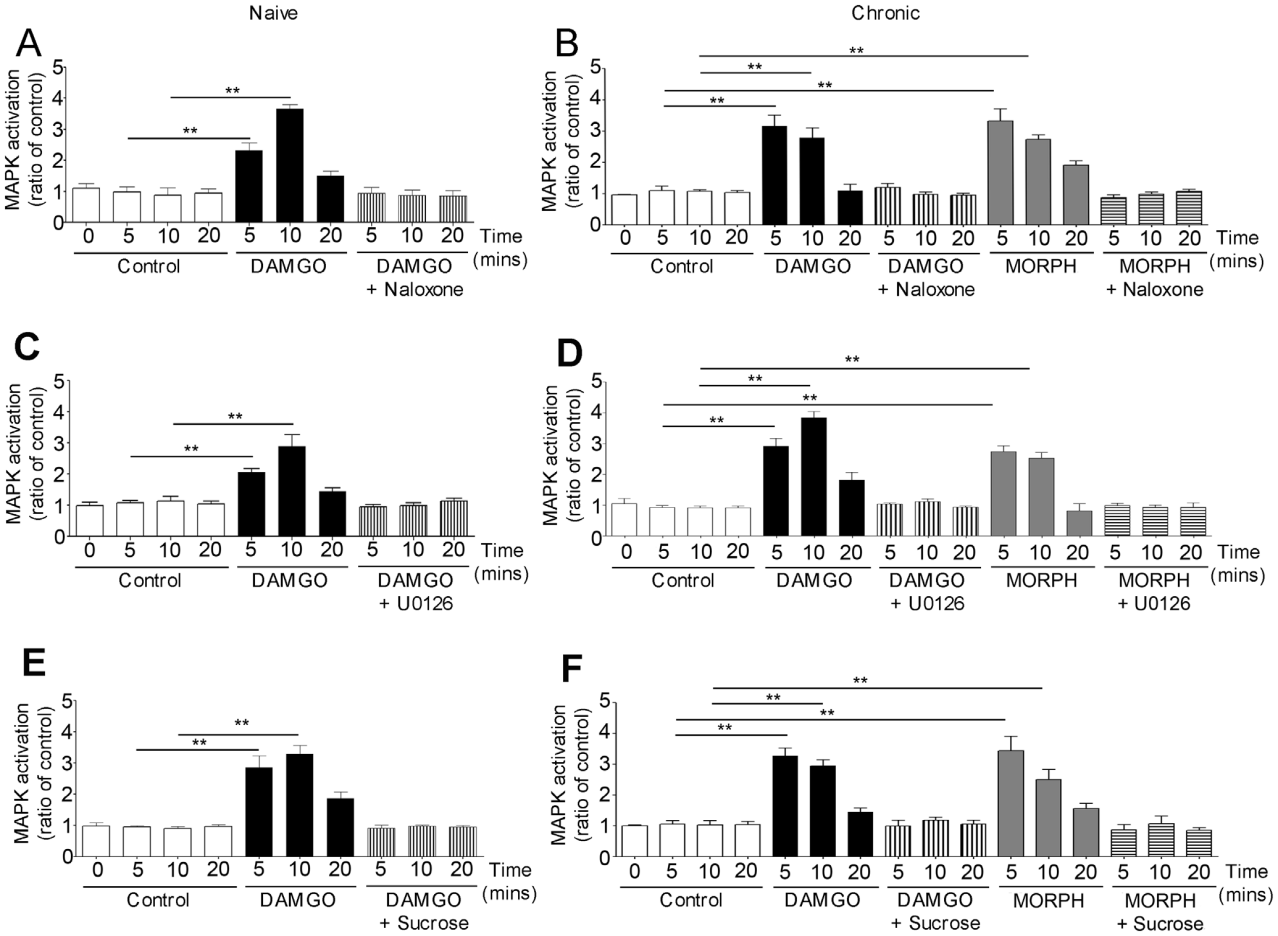
Characterization of opioid-induced MAPK pathway in enteric neurons. DAMGO-induced MAPK activation in naive (A, C, E) and DAMGO- and morphine-activation of MAPK in chronic (B, D, F) enteric neurons. Naloxone (A, B), MEK1/2 inhibitor, U0126 (C, D) and hypertonic sucrose (E, F) prevented opioid induced MAPK activation in naïve (A, C, E) and chronic (B, D, F) neurons. **p<0.01 significantly different from control. N = 4–7 experiments in triplicate per group.

Naloxone prevented opioid-induced ERK1/2 phosphorylation in naïve and chronic enteric neurons (Figure 3 A, B). Regardless of opioid stimulation, ERK activation in acute and chronic enteric neurons was unaffected by the sodium channel blocker TTX, indicating it was not due to endogenous opioid release (data not shown).

ERKs are the direct downstream proteins activated by MEK via phosphorylation [7]. To characterize the pathway of opioid-induced ERK1/2 activation, we pretreated naïve and chronic enteric neurons with a MEK1/2 inhibitor (U0126) prior to opioid stimulation. There was no ERK1/2 activation in DAMGO- and morphine-stimulated enteric neurons pretreated with U0126, supporting that opioid-induced ERK1/2 activation is MEK1/2 dependent (Figure 3 C, D).

**Figure 4:**
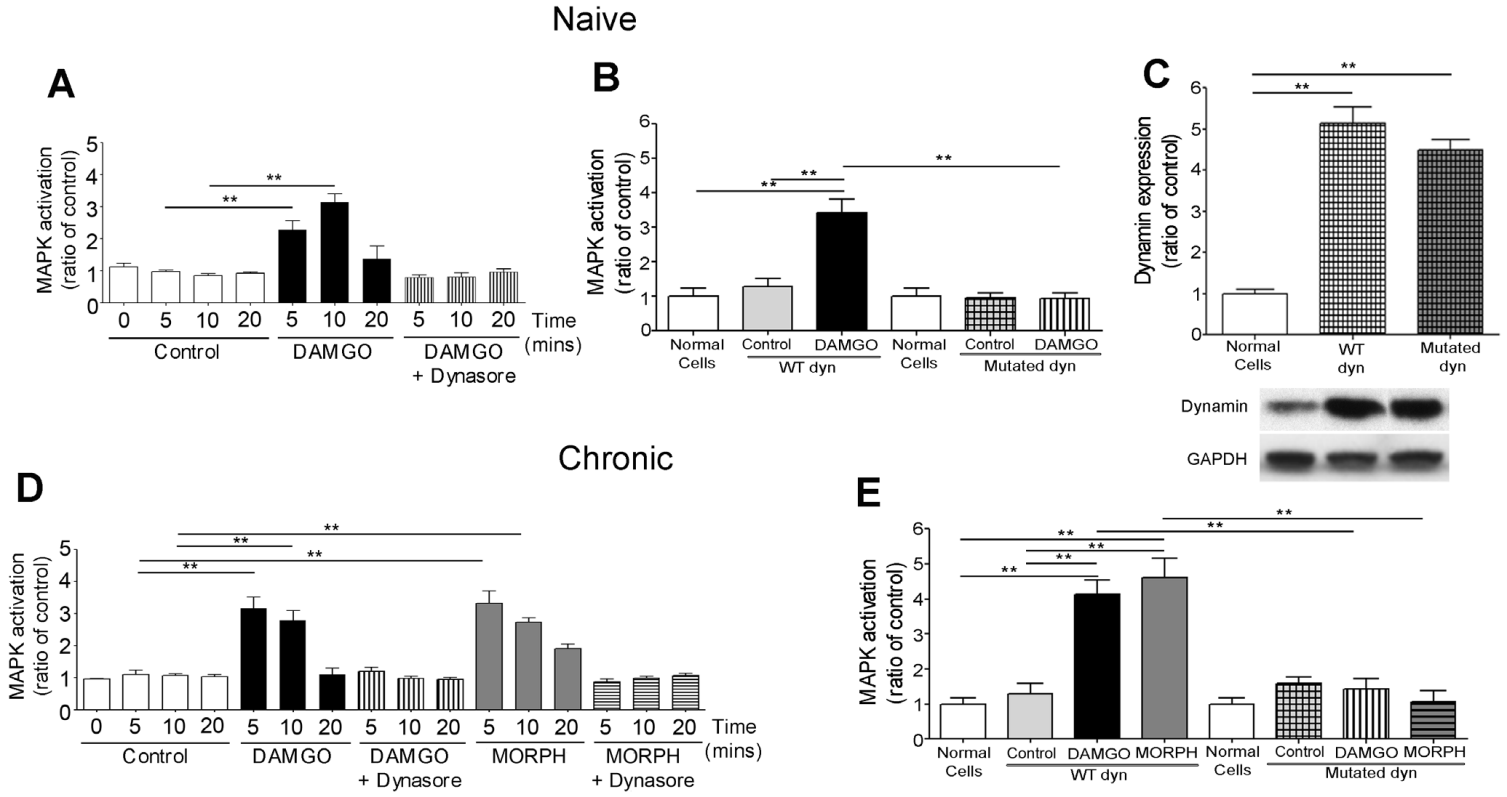
Role of dynamin on MAPK activation in enteric neurons. DAMGO-induced MAPK in naïve neurons is inhibited by dynasore, a dynamin inhibitor (A) and it is not observed in neurons transfected with mutated K44E dynamin (B). Both DAMGO- and morphine-induced MAPK activation in chronic neurons is blocked by dynasore (D) and is not detected in neurons transfected with mutated dynamin (E). C shows the increased expression of dynamin (dyn) immunoreactivity in enteric neurons transfected with wild type (WT) or mutated dynamin, confirming effectiveness of neuronal transfection. **p<0.01 compared to controls; n = 5–7 performed in triplicate per group. Representative immunoblots of dynamin immunoreactivities are shown at the bottom of histogram in C. GAPDH served as housekeeping protein to verify that the same amount of proteins was loaded on each gel.

Opioid-induced ERK1/2 activation in enteric neurons was blocked by pretreatment with hypertonic sucrose solution (Figure 3 E, F) and with the dynamin inhibitor, dynasore (Figure 4 A and D). Furthermore, ERK1/2 phosphorylation was observed in opioid-stimulated enteric neurons transfected with WT dynamin, but not in those expressing the mutated dynamin (Figure 4 B, E). Western blot analysis showed a significant increase (p<0.01) in the expression of dynamin in enteric neurons transfected with either WT or mutated dynamin compared to non transfected neurons, showing that transfection was effective (Figure 4 C). These findings confirmed that opioid-induced ERK1/2 activation in enteric neurons is endocytosis- and dynamin-dependent.

### Desensitization of opioid-induced ERK activation

**Figure 5:**
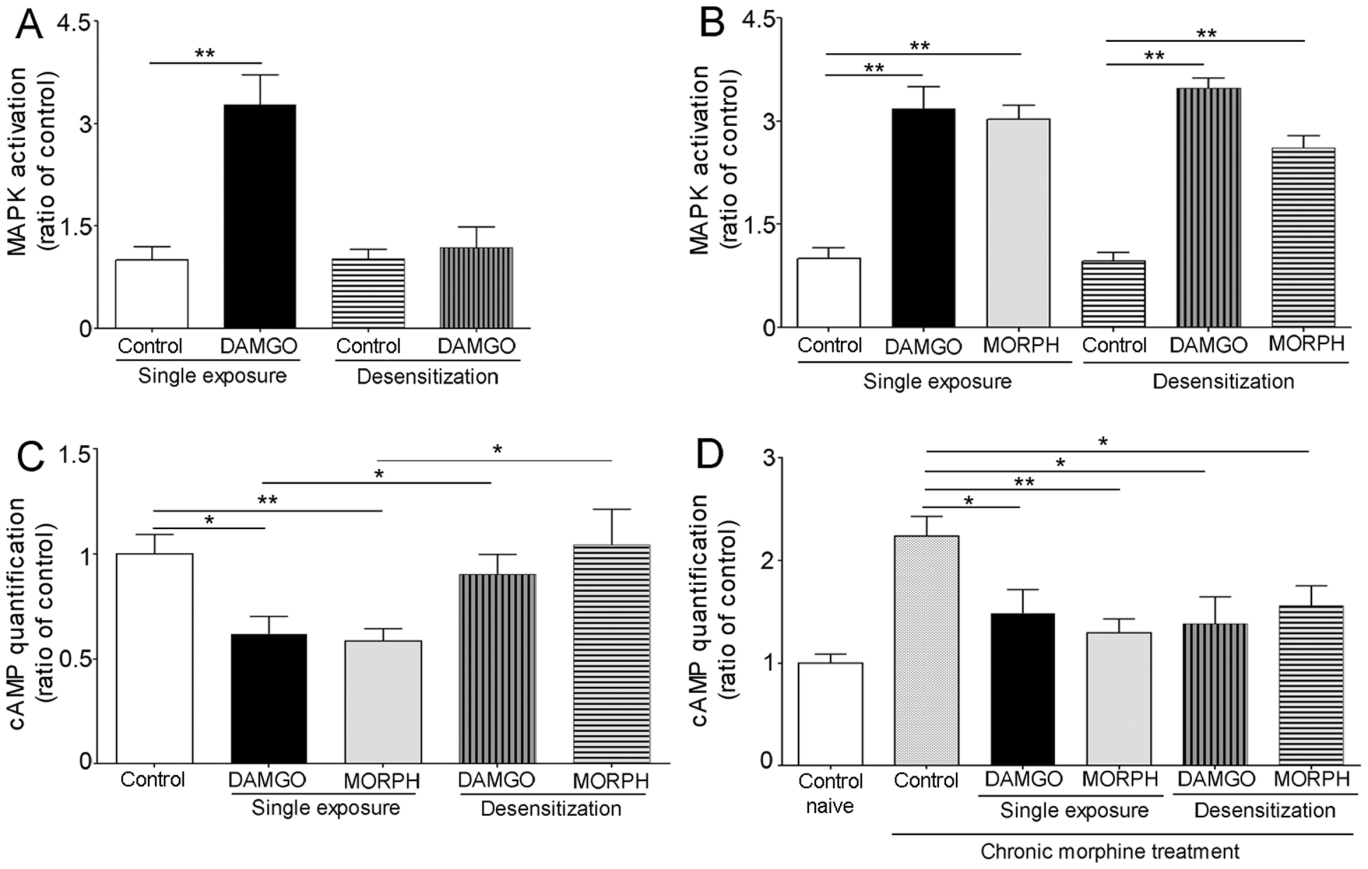
Desensitization of μOR signaling in enteric neurons. A: Single exposure to DAMGO (1 µM, 5 min) induced significant MAPK activation in naïve enteric neurons, whereas a second exposure to the same DAMGO dose following 2 hours DAMGO pretreatment abolished DAMGO-mediated MAPK response, indicating desensitization. B: Single exposure to DAMGO (1 µM) or morphine (10 µM) activated MAPK in chronic neurons. A second exposure to the same dose of DAMGO or morphine following 2 hours DAMGO or morphine pretreatment induced the same effect in chronic neurons as single exposures, indicating suppression of desensitization. (** p<0.01 vs. control in A and B). C and D: DAMGO and morphine inhibit forskolin-stimulated cAMP in naïve (C) and chronic (D) enteric neurons. This effect was not observed in naïve enteric neurons (C) with a second opioid stimulation following a prior 2 hour exposure, indicative of desensitization. D: Note the over 2 fold increase in cAMP in unstimulated chronic neurons (cAMP superactivation or “overshooting”) vs. naïve control; DAMGO and morphine inhibition of cAMP was not prevented by 2 hours DAMGO or morphine pretreatment in chronic neurons, indicating suppression of desensitization. **p<0.01 vs. controls. N = 5–7 experiments performed in duplicate per group.

In naïve enteric neurons exposed to DAMGO (1 µM) for 2 hours, a second exposure to the same concentration of DAMGO did not induce ERK1/2 phosphorylation suggesting desensitization of MAPK signaling (Figure 5 A). By contrast, in chronic enteric neurons stimulated with DAMGO (1 µM) or morphine (10 µM) for 2 hours, a second exposure to either DAMGO or morphine (at the same concentration) induced significant ERK1/2 phosphorylation, indicating suppression of desensitization of MAPK signaling (Figure 5 B). This prolonged signaling could be the initiating factor leading to cellular adaptations induced by prolonged opioid stimulation.

### cAMP signaling in enteric neurons and desensitization of cAMP inhibition

In naïve neurons, both DAMGO and morphine inhibit forskolin-stimulated cAMP. When naïve neurons were exposed for 2 hours to either DAMGO (1 µM) or morphine (10 µM), then stimulated again with the same opioid at the same concentration, there was no inhibition of cAMP indicating desensitization (Figure 5 C). In chronically treated enteric neurons, there was cAMP superactivation, which was inhibited by either DAMGO or morphine. Opioid-inhibition of forskolin-stimulated cAMP persisted in neurons exposed to a second dose of DAMGO or morphine following a previous incubation with the same agonist for 2 hours indicating loss of desensitization in chronic enteric neurons (Figure 5 D).

### ERK activation mediated CREB phosphorylation in enteric neurons chronically treated with morphine

**Figure 6:**
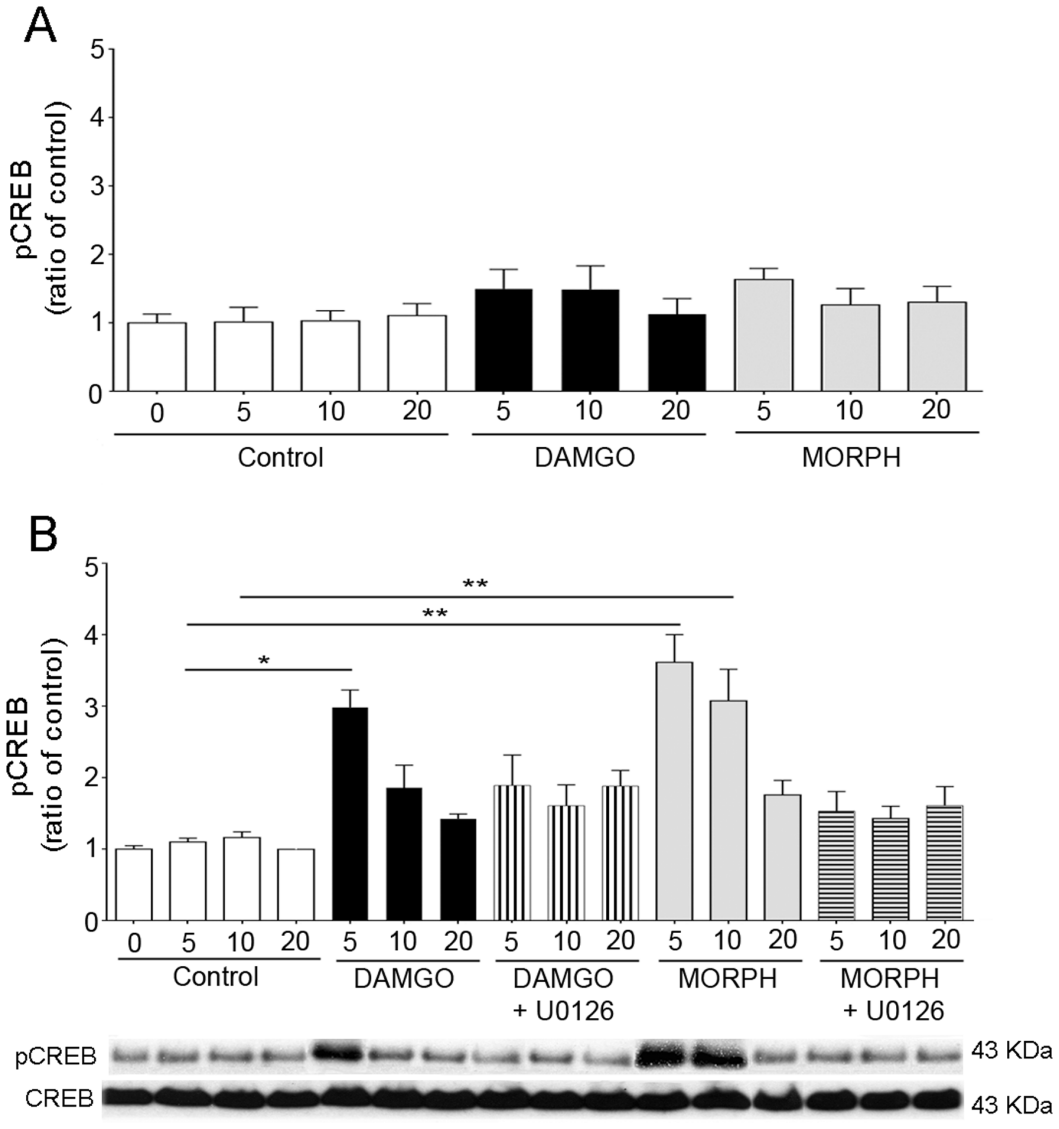
Effect of opioids on CREB phosphorylation in enteric neurons. Naïve (A) and chronic (B) neurons were stimulated with 1 µM DAMGO, 10 µM morphine or medium (control) for 0–20 minutes. DAMGO and morphine induced a significant, transient CREB activation in chronic, but not naïve enteric neurons. CREB phosphorylation in chronic neurons was blocked by the MEK1/2 inhibitor (U0126) treatment. *p<0.05 and **p<0.01 vs. control; n = 4–7 experiments in triplicate per group. Representative gels of pCREB and CREB are shown at the bottom of the figure. Total CREB was used to verify that the treatment did not affect the total level of this protein and to confirm equal gel loading.

DAMGO and morphine induced significant CREB phosphorylation at 5 and 10 minutes in enteric neurons chronically treated with morphine compared to controls, but not in naïve neurons (Figure 6). Opioid-induced CREB phosphorylation was abolished by MEK1/2 inhibitor treatment (Figure 6), indicating that CREB activation is MAPK/ERK pathway-dependent.

### Effect of MAPK pathway disruption on chronic morphine-induced GI transit delay

**Figure 7:**
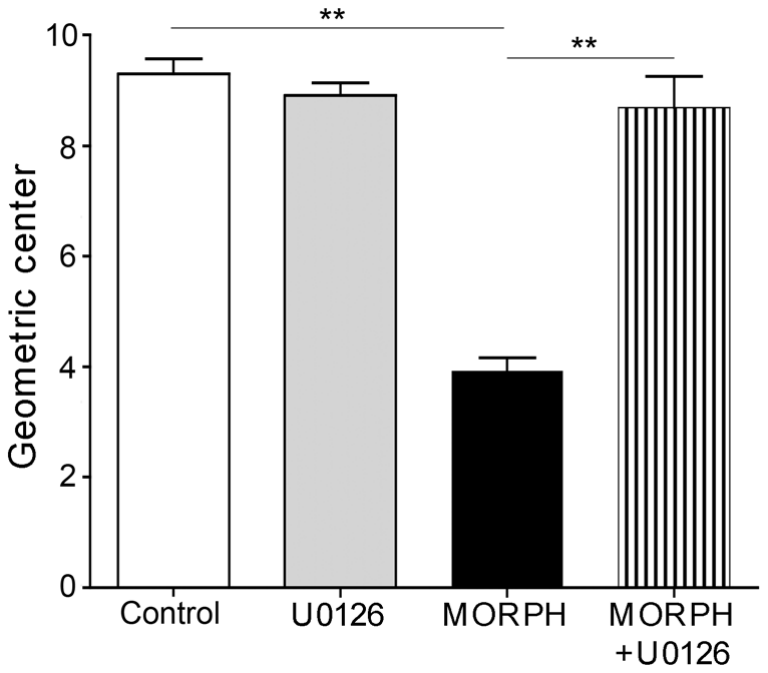
Effect of MEK1/2 inhibitor (U0126) on GI transit delay induced by chronic morphine in rats. Chronic morphine (MORPH) significantly delayed GI transit (reduction of geometric center) (**p<0.01 vs. control). MEK1/2 inhibitor alone (U0126) did not affect transit. Administration of MEK1/2 inhibitor together with morphine (MORPH+ U0126) reversed the delay in GI transit (**p<0.01 vs. MORPH). N = 5 per group.

We tested whether there was a correlation between MAPK signaling and functional response of GI transit to chronic morphine treatment. The pronounced delay of GI transit observed in rats chronically treated with morphine, was reversed to normal in rats chronically treated with morphine and MEK1/2 inhibitor (Figure 7). These data suggest that MAPK/ERK pathway plays a role in the opioid-induced impairment of GI motility, which is likely to involve CREB phosphorylation.

## Discussion

This study showed that opioids induce activation of MAPK/ERK1/2 in enteric neurons of the small intestine through a pathway requiring μOR internalization, whereas opioid inhibition of cAMP signaling is independent of ligand internalization efficiency. Chronic morphine treatment suppressed opioid-induced desensitization of cAMP and MAPK signaling and induced activation of the transcription factor, CREB through a MAPK/ERK pathway, since it was prevented by a selective MEK inhibitor, an upstream protein of ERK. Finally, MAPK/ERK blockade reversed total GI transit delay induced by chronic morphine treatment. These findings provide novel information on μOR signaling in rat enteric neurons, which mediate opioid-induced GI effects [4], [5], and suggest that prolonged μOR signaling and MAPK/ERK activation induced by long-term opioid treatment contribute to the development of opioid-induced constipation and are likely to involve CREB phosphorylation.

Several lines of evidence support the internalization-dependency of opioid-mediated MAPK/ERK activation in enteric neurons of the small intestine. Significant ERK1/2 phosphorylation was observed only in conditions in which opioids triggered endocytosis and was blocked by hypertonic sucrose, or dynasore, a dynamin inhibitor, and in enteric neurons transfected with a dominant negative K44E mutant dynamin, which prevent clathrin-dependent, dynamin-mediated endocytosis [13], [22]–[24], [31]. Our data in enteric neurons are consistent with previous reports of endocytosis as a prerequisite for opioid-induced MAPK activation in cell lines [27], [32], but in contrast with other studies indicating internalization-independence [33], [34], but dynamin-dependence [33], of opioid-stimulated MAPK pathway in heterologous cells and neuronal cell lines expressing endogenous μORs. Furthermore, the failure of morphine to activate ERK1/2 in naïve enteric neurons is consonant with findings in cultured striatal neurons [19], whereas in naïve neurons of the midbrain (Duraffourd and Sternini, unpublished) and in cell lines, morphine was effective in activating MAPK/ERK1/2 [27], [32]–[34]. Similarly, opioid internalization efficiency varies in different cell populations. Indeed, morphine fails to internalize μORs in enteric neurons of the small intestine, dorsal root ganglion cells, and subpopulations of CNS neurons, whereas it elicits internalization in other neuronal populations [11]–[13], [15], [16], [35]. Furthermore, chronic treatment with morphine reverses the resistance of morphine-activated μORs to internalize in enteric neurons of the small intestine *in vivo* [16] and *in vitro* (this study). Moreover, prolonged (2–24 hours) morphine stimulation results in μOR internalization in cultured dorsal root ganglion cells [35], but not in heterologous cell lines [36]. Taken together, these findings support the notion of cell specific μOR trafficking and signaling.

To determine μOR desensitization in naïve enteric neurons, we used the cAMP assay, since cAMP signaling is internalization-independent [37]. These studies showed that morphine induces the same level of desensitization of cAMP inhibition in enteric neurons as DAMGO, extending previous observations of morphine-induced desensitization in absence of internalization in different cell types [38], [39], indicating dissociation between internalization and desensitization [33], [38]. Furthermore, chronic morphine treatment suppresses μOR desensitization in enteric neurons as shown by the inability of opioids to activate MAPK/ERK and to inhibit cAMP, whereas it does not affect the magnitude of ligand-induced μOR internalization. This further supports the dissociation between these two regulatory processes, unlike previous findings in cell lines reporting that chronic morphine inhibits both opioid receptor desensitization and internalization [40]. Chronic morphine treatment does not affect opioid-evoked functional response in enteric neurons, as indicated by the same magnitude of opioid-induced μOR internalization and the same degree of MAPK/ERK activation and cAMP inhibition by opioids in naïve and chronic enteric neurons.

Together, these findings challenge the concept that morphine's inefficiency to internalize and desensitize μORs serves as the initial step to promote intracellular mechanisms, at least in enteric neurons of the small intestine, leading to long-term opioid side effects [6], [41], whereas receptor endocytosis would prevent compensatory downstream changes by limiting signaling [33]. Other mechanisms are likely to be involved in the development of long-term opioid side effects, including changes of G protein levels, trafficking of proteins and kinases, which would alter downstream signaling and resensitization [42]. We investigated whether in enteric neurons, opioids activate the transcription factor CREB, which has been shown to be involved with opioid-induced effects [7], [18], [19]. Significant increase in CREB phosphorylation was observed in chronically treated neurons in response to either DAMGO or morphine compared to naïve neurons and was prevented by MEK inhibitor. The activation of the transcription factor CREB in enteric neurons chronically stimulated with opioids is in agreement with previous studies in different types of neurons [7], [18], [19] and is likely to represent one of the intracellular adaptations induced by prolonged activation of μORs, though the exact mechanism behind this change remains to be fully elucidated. Activated ERK1/2 has different intracellular localization, which might vary according to the activation pathway and downstream cascade [43]. Studies in different types of cells including primary CNS neurons have shown that morphine activates ERK via protein kinase C (PKC) pathway and morphine-induced ERK phosphorylation remains in the cytosol inducing CREB, whereas internalizing opioids such as etorphine and fentanyl activate ERK in a ß arrestin-dependent pathway resulting in ERK nuclear translocation and activation of G protein kinase2 and ß arrestin2 transcription [43]. In our study, both morphine and the internalizing opioid, DAMGO induce internalization- and dynamin-dependent ERK phosphorylation and CREB phosphorylation in chronic neurons. However, ERK compartmentalization as well as the relative role of PKC or ß-arrestin in ERK activation were not investigated and further studies are required to determine the mechanism underlying CREB phosphorylation induced by chronic opioid treatment.

μOR enteric neurons are a heterogeneous population comprising ascending and descending neurons, interneurons and motor neurons [17]. μORs are Gi/Go coupled and when activated inhibit neurotransmitter release by modulating neuronal excitability [4], [5] in different neuronal pathways thus impairing motility and transit. Prolonged opioid exposure induces inhibition of neurotransmitter release thus impairing intestinal peristalsis and propulsion and inhibition of intestinal secretion further exacerbating GI transit delay [4], [5], [44], [45]. Our study shows that blocking the MAPK/ERK1/2 pathway and CREB activation with a selective MEK1/2 inhibitor reversed the GI transit delay induced by chronic morphine, suggesting that MAPK/ERK pathway plays a role in motility impairment resulting in constipation, which is likely to involve CREB. Since upregulation of CREB phosphorylation has been shown to affect neuronal excitability in CNS and ENS neurons [46], [47], it is tempting to speculate that activation of CREB contributes to GI transit delay by altering the neuronal circuits mediating GI motility. However, this remains to be elucidated. Finally, further studies are required to determine whether μOR signaling differs in enteric neurons of the colon compared to the ileum. This is likely based on previous findings of different functional responses of the ileum and colon following chronic morphine as indicated by the reduction of the neurogenic response of neuromuscular preparations of the ileum but not colon, which involves ß arrestin [48], [49].

In summary, this study shows that opioid activation of MAPK/ERK signaling pathway in enteric neurons is endocytosis- and dynamin-dependent, whereas cAMP signaling is inhibited by opioids irrespective of their internalization efficiency. Chronic morphine treatment suppressed μOR desensitization thus resulting in prolonged signaling, and induced MAPK/ERK-dependent activation of the transcription factor, CREB. Opioid-induced CREB upregulation is likely due, at least in part, to cytosolic ERK since it was observed only in neurons chronically treated with morphine and morphine-activated ERK has been reported to be retained in the cytosol [43], though the contribution of nuclear ERK cannot be discounted at this point. Finally, the prevention of morphine-induced delay of GI transit by blockade of MAPK/ERK/CREB cascade in enteric neurons strongly supports the involvement of this downstream signaling pathway in the development of opioid-induced constipation in chronic conditions, a major limiting factor in the use of opioids in pain management. Intervention at the level of the μOR-mediated activation of the MAPK/ERK signaling pathway in enteric neurons might provide a novel, potential therapeutic target for opioid-induced GI dysfunction following chronic opioid exposure.
